# Construction and Rescue of a Molecular Clone of Deformed Wing Virus (DWV)

**DOI:** 10.1371/journal.pone.0164639

**Published:** 2016-11-09

**Authors:** Benjamin Lamp, Angelika Url, Kerstin Seitz, Jürgen Eichhorn, Christiane Riedel, Leonie Janina Sinn, Stanislav Indik, Hemma Köglberger, Till Rümenapf

**Affiliations:** 1 Institute of Virology, Department of Pathobiology, University of Veterinary Medicine, Vienna, Austria; 2 Institute of Pathology and Forensic Veterinary Medicine, Department of Pathobiology, University of Veterinary Medicine, Vienna, Austria; 3 Institute for Apiculture, Agricultural Inspection Service and Research Centre Vienna, Austrian Agency for Health and Food Safety and Federal Office for Food Safety, Vienna, Austria; University of Salford, UNITED KINGDOM

## Abstract

European honey bees are highly important in crop pollination, increasing the value of global agricultural production by billions of dollars. Current knowledge about virulence and pathogenicity of *Deformed wing virus* (DWV), a major factor in honey bee colony mortality, is limited. With this study, we close the gap between field research and laboratory investigations by establishing a complete *in vitro* model for DWV pathogenesis. Infectious DWV was rescued from a molecular clone of a DWV-A genome that induces DWV symptoms such as crippled wings and discoloration. The expression of DWV proteins, production of infectious virus progeny, and DWV host cell tropism could be confirmed using newly generated anti-DWV monoclonal antibodies. The recombinant RNA fulfills Koch’s postulates circumventing the need of virus isolation and propagation of pure virus cultures. In conclusion, we describe the development and application of a reverse genetics system for the study of DWV pathogenesis.

## 1. Introduction

The Western or European honey bee (*Apis mellifera*, Linnaeus 1756) is a eusocial insect living in colonies with three distinct castes: a single female queen, male drones, and female workers. Bee products like honey, wax, royal jelly or propolis are important to mankind [1]. Insect pollination is essential to agricultural productivity and human nutrition [2]. The pollination service of honey bees on crops, such as fruit trees and berries, in global agriculture has been estimated to be worth around €100 bn per year [3, 4].

Beekeepers have been reporting on unusually high honey bee colony mortality in North America [5] and Europe [6] for more than a decade. Natural populations of European honey bees are threatened or already extinct in many parts of the world. Parasitic infections cause a decline of feral honey bee colonies even in their original habitats [7]. Colony mortality is associated with strong *Varroa destructor* mite infestation and a rapid decrease of the adult bee population [8, 9]. The mite *Varroa jacobsoni*, a parasite of the Eastern honey bee (*A*. *cerana*), adapted to *A*. *mellifera* in the 1950s giving rise to the new species *V*. *destructor* [10]. Due to colony and queen bee trade, *V*. *destructor* has spread to all continents with the exception of Australia [11]. The global invasion reached central Europe in the 1970s and the USA in the 1980s [8]. Nowadays, bee colony survival depends on human supervision, intervention and regular control measures such as biotechnical or miticide treatments [12, 13].

The occurrence of deformed emerging bees is the major clinical sign of varroosis [14]. These deformities were closely associated with DWV infections as previously shown using experimental infection of pupae [15]. DWV is present in almost every beehive throughout the year, but DWV symptoms only occur in heavily Varroa infested colonies [16]. Overt DWV infections, characterized by the appearance of DWV symptoms at a colony level, are indicative of a colonies downfall and occur primarily during autumn [17]. There is general consensus that a synergistic interaction between DWV and Varroa mites plays an important role in colony mortality [18–21]. The Varroa mites’ feeding behavior transmits DWV directly into the bees’ haemolymph [16, 22, 23], bypassing the natural barriers of the bees body against conventional vertical and horizontal routes of virus transmission [24, 25].

It was documented that diverse DWV strains were present in the honey bee population of Hawaii before the Varroa mite invaded these islands, whereas a single strain prevails after the mite invasion [18]. A recent phylogenetic analysis supports the hypothesis that a DWV epidemic started in the mid of the 20^th^ century coinciding chronologically with the Varroa mites’ host switch to *A*. *mellifera* [26]. DWV belongs to the genus *Iflavirus* within the family *Iflaviridae*. Iflaviruses are members of the order *Picornavirales* with a Picornavirus-like genome organization and particle morphology [14]. DWV is separated into the three master variants DWV-A, -B and -C [27]. Master variant A contains the classical DWV strains [28] and Kakugo virus [29], which are responsible for elevated colony mortality [18]. DWV-B includes the strains of *Varroa destructor viruses* (VDV), which are able to protect colonies by superinfection exclusion against DWV-A [30]. High recombination frequencies between different DWV strains and master variants have been documented in the field [31, 32]. However, only a few genomic sequences of DWV field strains have been deposited in databases and recombination was not proven *in vitro* using clonally selected viruses.

DWV has a single-stranded RNA genome of about 10 kb. The genome is polyadenylated and contains a single open reading frame (ORF) that is flanked by non-translated regions (NTRs). The ORF encodes a 2,894 amino acid polyprotein, which is translated via an internal ribosomal entry site (IRES). Viral proteases mediate maturation of at least four structural proteins (VP1-4) from the N-terminal third of the polyprotein and of a replicase unit (RNA helicase, 3C protease, and RNA-dependent RNA polymerase) from the C-terminal part. A genomic sequence of DWV is known since 2006 [28] and plasmids containing DWV genomes have been presented [33]. The IRES element of DWV has been studied in detail documenting its functional activity [34, 35].

Only a limited number of virological tools have been developed for the laboratory research on bee viruses. Defined cell culture systems, serological reagents, characterized viral strains or clones are effectively missing despite decades of intense research. So far, all studies used bee models and virus isolates originating directly from the field or from propagation in bee infection models. The use of virus isolates from field samples, which had not been plaque purified in cell culture (biological cloning), may result in misleading conclusions. Multiple virus strains, master variants or even species could be present at the same time in such field isolates. The application of reverse genetics allows circumventing virus isolation, clonal plaque purification and production of clonal viruses in cell culture systems. To our best knowledge, no reverse genetics system has been presented for DWV or any other member of the genus *Iflavirus*. For bee viruses in general, a single publication reported on the generation of infectious transcripts of *Black queen cell virus* (BQCV) achieved by the assembly of full-length PCR products with a suitable recognition site for the T7 RNA polymerase [36]. Here, we describe a stable infectious plasmid clone of DWV and its application for studies on DWV pathogenesis.

## 2. Materials and Methods

### 2.1. Field sample

The DWV-positive bee sample used in this study originated from a colony loss in Austria that occurred in 2012. No specific permission was required for honey bee sample collection, because this study used remnants of specimens initially collected from colony losses for diagnostic purposes. Crude bee lysate from the field sample was injected into the ventral chest of white to purple eye honey bee pupae (13 to 15 days old) to propagate DWV. The infected bee pupae were incubated for three days at 35°C in a humid environment. All experiments with infectious DWV were carried out in biosafety level 2 facilities.

### 2.2. Stock preparation and virus purification

Single pupae were snap-frozen in 1 ml phosphate buffered saline (PBS) and homogenized in a TissueLyser II (Qiagen, Hilden, Germany). The lysate was cleared by centrifugation (13,000 rpm for 1 min) and filtered through a 0.2 μm nylon filter membrane (Acrodisc, Sigma-Aldrich). Viral load was measured as genome equivalents (GE) using quantitative RT-PCR (qRT-PCR). For concentration and purification, the filtrate was centrifuged through a 2 ml cushion of 50% sucrose (w/v) in a Beckman centrifuge (36,000 rpm, 4°C, 4 h, SW41 Ti). The resulting pellet was resuspended in 100 to 200 μl PBS and again, cleared by centrifugation (13.000 rpm for 1 min). All virus stocks were tested for the presence of *Acute bee paralysis virus* (ABPV) and *Sacbrood virus* (SBV) by conventional RT-PCR [37, 38].

### 2.3. RNA purification and RT-PCR

RNA was extracted from whole pupae lysates or concentrated virus suspensions employing the QIAamp viral RNA Mini Kit (Qiagen, Hilden, Germany) and eluted in 60 μl nuclease free water. RT-PCR was carried out using the Long Amp RT-PCR Kit or the OneTaq One-Step RT-PCR Kit (NEB, Ipswich, USA). For 5’-RACE-PCRs, the DWV concentrate was digested with proteinase K prior to RNA extraction. A classic 5'-RACE protocol was adapted using the Long Amp RT-PCR Kit. Briefly, first strand cDNA was synthesized from total RNA using the DWV specific primer PDV16 and precipitated with ethanol. Poly-A or poly-C tailed cDNA was generated using terminal deoxytransferase (TdT; NEB, Ipswich, USA). Another genome specific primer (PDV21) was used in conjunction with a primer containing oligo-dt / oligo-dg and adapter sequences (PDV18/PDV19) to amplify the DWV 5’-end. Finally, a nested PCR was performed using primers hybridizing with adapter (PDV20) and 5’-terminal DWV sequences (PDV22).

### 2.4. Generation of a full-length DWV-A clone

The DWV-A field isolate 1414 was chosen for our molecular cloning attempts because passaging of this virus yielded deformed or dead bees containing high DWV titers. In addition, this field sample did not contain ABPV or SBV. Sequence alignments of four DWV-A isolates (AJ489744, AY292384, JQ413340, AB070959) from databases allowed the design of DWV-A specific oligonucleotides (Table 1). The overlapping RT-PCR fragments A (PDV1 and PDV16) and B (PDV5 and PDV18) were directly analysed by Sanger sequencing (Eurofins Genomics, Vienna, Austria) applying oligonucleotides PDV1 to PDV18 to assemble a master-sequence of DWV-A 1414. T-vector clones (Promega, Fitchburg, WI) containing the 5’-RACE-PCR products were sequenced with M13forw and M13rev primers. Based on the sequence information, a full-genome RT-PCR was designed. The complete DWV genome was amplified using the OneTaq One-Step RT-PCR Kit, oligonucleotides PDV17 and PDV23, and RNA purified from a virus concentrate (1,8 x 10^11^ GE). The 10,164 bp RT-PCR product was inserted in a pBR322 derived vector using a Gibson assembly reaction [39]. This vector already contained all features necessary for RNA translation providing an SP6 promoter, a poly-A sequence and a NotI site for linearization. For differentiation of cloned recombinant DWV (rDWV) and DWV field strains, a novel BamHI site at nt position 2,851 was introduced using a Q5 extension PCR (PDV24 and PDV25) preserving the encoded amino acid sequence. The plasmid (pL427) was sequenced after two passages in bacteria (*E*. *coli*, strain HB101) to confirm sequence stability. With the exception of several synonymous mutations within the coding region, no differences were found between the DWV-A 1414 master sequence and the cloned rDWV sequence.

**Table 1:** Oligonucleotides used in this study—nt position refers to DWV, strain Austria 1414 (KU847397).

Name	Sequence	nt Position
PDV1	cgatttatgccttccatag	forw. 22–40
PDV2	gagctgggacccctcagtctc	forw. 766–786
PDV3	gtgttgcaactcgcttcgttc	forw. 1583–1603
PDV4	gaggatttgaatatatcgtc	forw. 2230–2249
PDV5	gtggttcattagaatatag	forw. 2975–2993
PDV6	ctgctaatcaacaaggacctg	forw. 3746–3766
PDV7	gtctagcgctgcatctagttatg	forw. 4434–4456
PDV8	ctactgtagattttagtaataattg	forw. 5153–5177
PDV9	gatcgtattgctatggaagc	forw. 5911–5930
PDV10	caagctccaagaaatcctgatg	forw. 6559–6580
PDV11	gataagtatttaactcgtcccgtg	forw. 7234–7257
PDV12	gagtgttagtaactggcgac	forw. 7946–7965
PDV13	gatatcttggaatactagtgctg	forw. 8637–8659
PDV14	ctgatttgcctttgtccgag	forw. 9359–9378
PDV15	cacatgggaagaaatggatg	forw. 9807–9826
PDV16	gtaaatcaaatactacataactc	rev. 3105–3127
PDV17	actactatggttaaaactatac	rev. 10143–10164
PDV18	ggccacgcgtcgactagtacttttttttttttttttt	rev. oligo-dt-adapter
PDV19	ggccacgcgtcgactagtacggggggggggggggggg	rev. oligo-dg-adapter
PDV20	ggccacgcgtcgactagtac	rev. adapter RACE
PDV21	gagactgaggggtcccagctc	rev. 766–786
PDV22	ctctactcgatactgcagtg	rev. 529–548
PDV23	tttaaaattcgctatgggagg	forw. 1–21
PDV24	gatcccttttgttacaattagatg	forw. 2852–2875
PDV25	ctttagcatgatctttcttc	rev. 2832–2851
PDV26	attgtgccagattggactac	forw. 2368–2387
PDV27	agatgcaatggaggatacag	rev. 2783–2802
PDV28	caagaattgtgccagattggactactg	forw. 2363–2389

### 2.5. RNA in vitro synthesis and transfection

Synthetic infectious RNA was produced as previously described [40]. Briefly, 2.5 μg DNA of the plasmid was digested with NotI and purified using phenol-chloroform extraction. The linearized plasmid DNA was transcribed into genomic rDWV RNA using SP6-polymerase (NEB, Ipswich, MA). 50 μl of the transcription mixture was DNase digested, RNA was purified with the RNeasy Mini Kit (Qiagen, Hilden, Germany) and eluted in 30 μl RNase free water. One microliter of the purified synthetic RNA (8.2 x 10^11^ GE) was injected in the chest of apparently healthy blue-eye pupae using a Hamilton syringe (Model 702). Mock infections with 1 μl of PBS and infections with 1 μl of a DWV-A 1414 virus stock (wtDWV, 8.0 x 10^7^ GE) were performed as controls. All pupae used in the experiment were extracted from the same comb. After injection, the pupae were transferred to a 24-well plate, incubated at 35°C for 3 days and harvested or left for further development until emergence.

### 2.6. Characterization of rDWV pathogenicity

Three bee pupae from a third passage of rDWV, wtDWV-A 1414, and negative controls were disrupted in 1 ml of PBS each. The pupae lysates were pooled for each inoculum and sterile filtered (pore size 0.2 μl) to prepare infectious stocks. Virus stocks were diluted with PBS to a final concentration of 1.0 x 10^10^ GE/ml. A calculation of the infectious dose (TCID_50_/ml or PfU/ml) was not possible due to the lack of suitable systems. 40 white to blue eye bee pupae (14 to 15 days old) were extracted from one comb. The pupae were incubated at 35°C over night to allow the detection of accidentally injured animals. The next day, groups of ten pupae were infected with equal doses (5.0 x 10^6^ GE) of rDWV and wtDWV by injection. The pupae were further incubated for six days until the end of development (day 22).

### 2.7. GE quantification and marker identification

An established diagnostic RT-PCR protocol [41] was adapted for GE quantification using PDV26 and PDV27. GEs were calculated by 7500 System SDS Software (Applied Biosystems, Foster City, USA) based on the standard curve of a cDNA plasmid. A different RT-PCR was used to determine the presence of the BamHI marker mutation in DWV genomes. A 764 nt fragment flanking the novel BamHI site was amplified using PDV16 and PDV28. Half of the PCR product was incubated with 20U BamHI for 30 min at 37°C and subjected to agarose gel electrophoresis. The appearance of two bands (488 and 276 nt) after BamHI digestion proved the presence of recombinant DWV.

### 2.8. Generation of mAbs against DWV VP1

**Fig 1:**
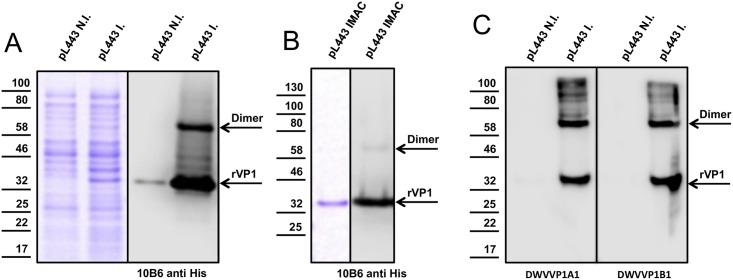
Recombinant DWV VP1 (rVP1) and VP1 specific antibodies. (A) rVP1 was expressed in *E*. *coli* via plasmid pL443. Total protein from non-induced (NI) and induced (I) cultures was separated by SDS-PAGE and either stained with Coomassie blue or probed with an anti His-tag antibody. (B) IMAC purified rVP1 revealed by Coomassie stain and western blot analysis. (C) Reactivity and specificity of two mouse monoclonal antibodies is shown using *E*. *coli* expressing rVP1. Arrows indicate the protein bands of rVP1 and rVP1 dimer.

The region coding for amino acid 622–894 of the DWV-A 1414 ORF was amplified by RT-PCR and inserted in a pet11a vector. The recombinant fragment of VP1 (rVP1, calculated molecular mass of 33 kDa) was prepared using heterologous expression in the *E*. *coli* strain Rosetta (Fig 1A). The histidine-tagged protein was purified by ion metal affinity chromatography (IMAC), as previously described [40]. Amount and purity of protein was determined by SDS-PAGE, and its identity was confirmed using an anti-His antibody (Fig 1B). BALB/c mice were immunized four times with rVP1 on days 0, 14, 28, and 42. Spleen cells were prepared and fused with sp2/0-AG14 myeloma cells to generate mAb producing hybridomas. Finally, 96 reactive mAbs were evaluated using ELISA, immunoblot (Fig 1C) and immunofluorescence assays against rVP1 and lysates of DWV infected bees. All animal use protocols employed in this study were approved by the institutional ethics and animal welfare committee and the national authority according to §§ 26ff. (Animal Experiments Act from 2012; BMWF-68.205/0107-II/3b/2013).

### 2.9. Western blot analysis

Protein from total pupa homogenate was precipitated by the addition of 4 volumes of ice-cold acetone for Western blot analysis. The pellet was resuspended in distilled water and boiled in sodium dodecyl sulfate-polyacrylamide gel electrophoresis (SDS-PAGE) buffer. Samples were separated on 7.5% acrylamide gels prior to electrophoretic transfer to nitrocellulose membranes (Pall corporation, Port Washington, New York). To detect DWV antigens, blots were blocked with 5% skim milk powder and probed with different mouse monoclonal antibodies against DWV VP1. The blots were developed using Amersham ECL plus reagent (GE Healthcare, Chalfont St Giles, GB) and exposed to a C-Digit scanner (Licor, Lincoln, Nebraska) and X-ray films for imaging.

### 2.10. Immunohistochemistry staining

Immunohistochemical investigations were performed using primary antibodies against DWV VP1 on an autostainer (Lab Vision AS 360) using the avidin-botin complex (ABC)-method. Briefly, 3 μm sections of fixed (4% formalin, 5% glycerin, 50% ethanol) and paraffin-embedded pupae were placed on coated slides and dried to enhance tissue adherence. The sections were deparaffinized in a descending alcohol series and rehydrated. Endogenous peroxidase activity was blocked by incubation in H_2_O_2_. The antigen-antibody-complex was detected using biotinylated anti-mouse IgG (Vector Laboratories, dilution 1:300), followed by incubation with streptavidin-peroxidase and visualization with diaminobenzidine (DAB; Labvision, Thermo Fisher Scientific). Subsequently, the sections were counterstained with haematoxylin, dehydrated and mounted.

### 2.11. Electron microscopy

Concentrated virus samples were adsorbed to glow discharged, carbon coated copper grids and negatively stained with 1% uranyl acetate. Samples were analysed on a FEI Morgagni 268D electron microscope equipped with an 11 megapixel CCD camera (Morada, Olympus SIS) at 80kV and 24,000x nominal magnification.

## 3. Results

### 3.1. Genome sequence of DWV-A strain 1414

**Fig 2:**
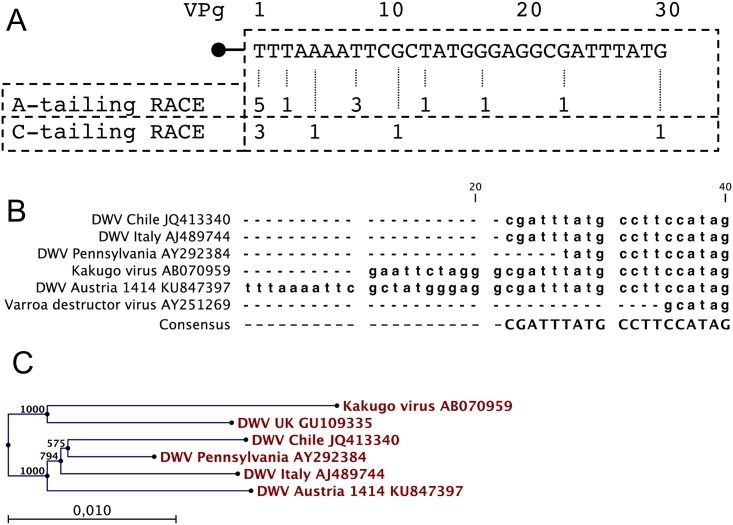
Sequence analyses of DWV-A 1414. (A) DWV 5’-NTR sequences were obtained from tailing RACE-PCRs. Numbers of clones containing the respective 5’-terminal nucleotides are indicated below the sequence. (B) Sequence comparison of the 5’-terminus of DWV-A 1414 and related Iflaviruses. Genbank entries are provided behind the strain designations. (C) Neighbor joining analysis using genomic sequences of different Iflaviruses. The phylogenetic analysis documents a close relationship of DWV-A 1414 to other DWV-A isolates from Europe and America. The number of substitutions per site is given as a scale and bootstrap values for 1,000 replicates were indicated above all nodes.

The complete genome of DWV-A 1414 (GenBank KU847397) was sequenced from RT-PCR fragments to establish an optimal genetic background for the development of a reverse genetics system. 5′ RACE-PCRs were performed, since remarkable size differences in the 5’-NTR between published DWV genomes were found. Amplicons from an A-tailing RACE and from a C-tailing RACE reaction were cloned and sequenced to uncover the ultimate 5’-end of DWV-A 1414. The majority of clones from both reactions contained a 5’-end sequence that was longer than any of the publicly available full-length DWV genome sequences (Fig 2A). As expected, most of the 5′-NTR was highly conserved between all DWV strains. However, a 5’-terminal stretch of 10 nucleotides was missing from Kakugo virus (DWV-A, AB070959) and at least 21 nucleotides were missing from all other DWV-A genomes and Varroa destructor virus 1 (DWV-B, AY251269, Fig 2B). A length difference of 14 nucleotides was observed, when comparing DWV-A 1414 (KU847397) to the recently published genomic sequence of DWV-C (ERS657949) [27].

**Fig 3:**
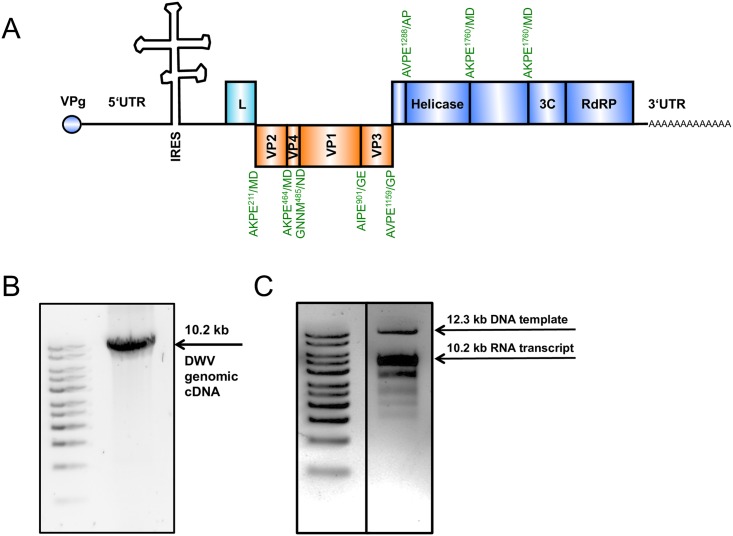
Molecular clone of DWV-A 1414. (A) Genome organization: VPg, 5’- and 3’-NTR, ORF, and poly-A tract are indicated. Within the ORF, the putative polyprotein cleavage sites are shown. Boxes mark annotated polyprotein genes or motives, with nonstructural proteins above and structural proteins below the line. (B) Genomic RT-PCR of DWV-A 1414. (C) In vitro transcription of rDWV-A 1414 RNA. Linearized plasmid DNA and genomic RNA transcript are indicated.

A phylogenetic analysis demonstrated the close relationship between published DWV-A sequences and our Austrian field strain from 2012 (Fig 2C). Pairwise identities to DWV-A 1414 are 98.0% for a DWV-A strain from Pennsylvania (AY292384), 97.6% for a DWV-A strain from Italy (AJ489744), 97.4% for a DWV-A strain from Chile (JQ413340.1), 96,7% for Kakugo virus (AB070959), but only 81.4% for Varroa destructor virus (DWV-B, AY251269.2). *In silico* translation revealed that all postulated 3C protease cleavage sites [28] were present in the polyprotein of strain A1414 (Fig 3A).

### 3.2. Rescue of recombinant DWV (rDWV)

**Fig 4:**
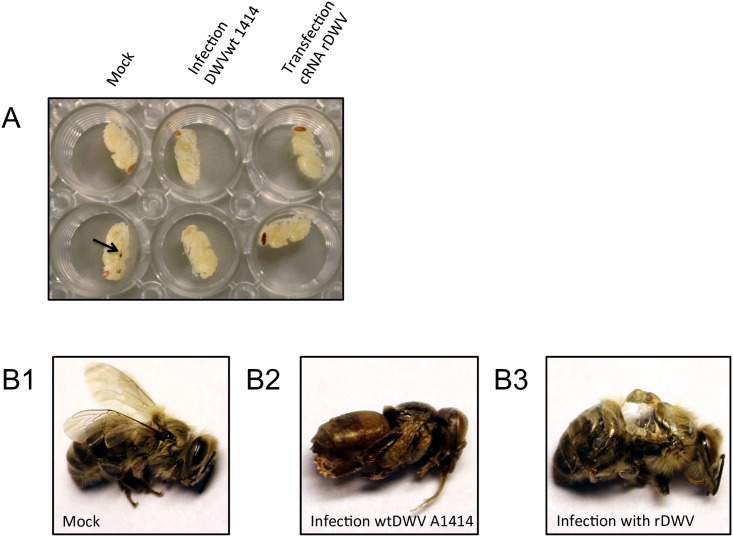
DWV infection model. (A) Bee pupae at the day p.i.. A black arrow at one bee pupa marks the injury caused by injection. (B) Typical outcome of infections with rDWV-A 1414 and wtDWV-A 1414: (B1) Healthy bee, which emerged at day 21 of development after mock infection. (B2) Bee pupa, which died three days after infection with wtDWV-A 1414. (B3) Nonviable adult bee, which emerged at day 21 after infection with rDWV-A 1414 showing typical wing and limb deformities.

**Fig 5:**
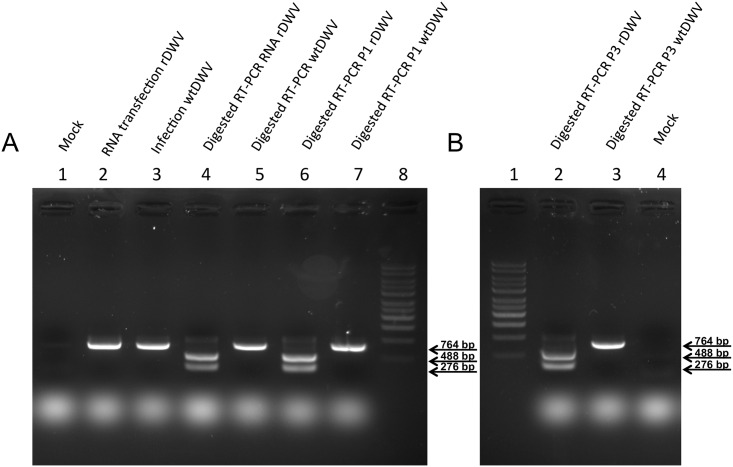
Differentiation of rDWV and wtDWV using the BamHI restriction site. Pupae transfected with synthetic RNA of rDWV-A 1414 (lane A2) and infected with wtDWV-A 1414 (lane A3) show the DWV specific RT-PCR product of 764 bp. Mock infected pupae (lane A1, B4) serve as negative controls. The PCR product of pupae transfected with rDWV-RNA (lane A4), infected with passage one rDWV (lane A6) and with passage three rDWV (lane B2) was converted in 488 bp and 276 bp fragments after BamHI digestion. In contrast, the PCR products of wtDWV infected controls (lane A5, A7, and B3) are not cleaved by BamHI. Arrows on the right side indicate the size of the respective visible RT-PCR products and fragments.

**Fig 6:**
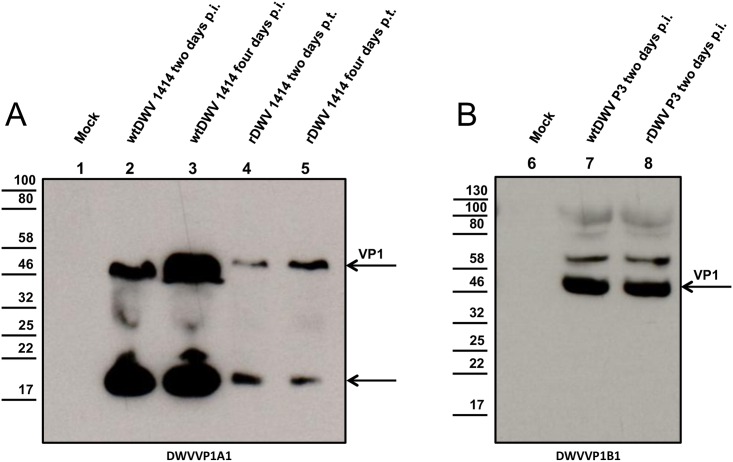
Western blot analysis of the rDWV rescue. (A) Bee pupae infected with a DWV-A field isolate (wtDWV, lane 2 and 3) or transfected with synthetic RNA of the DWV-A clone (rDWV, lane 4 and 5) were probed two days and four days after inoculation using mAb DVWVP1A1. Bands with an apparent molecular mass of 46 kDa (VP1) and 19 kDa (unknown) were visible in wtDWV infected and rDWV transfected pupae but absent in the mock control (lane 1). (B) Bee pupae infected with passage three of wtDWV (lane 7) and rDWV (lane 8) were tested for VP1 expression using mAb DVWVP1B1 two days after infection. Interestingly, the 19 kDa protein was not detectable using mAb DVWVP1B1.

A dose of 8.2 x 10^11^ GE synthetic RNA (Fig 3B and 3C) was injected in the thorax of blue eye bee pupae for the rescue of rDWV. Bee pupae showed a melanization at the injection site (Fig 4A, arrow). Following a three-day incubation, the bee pupae were homogenized in a total volume of 1 ml PBS. A mean viral load of 2.4 x 10^8^ GE/bee of rDWV was found in the transfected bee pupae using qRT-PCR. The rDWV was passaged in novel bee pupae using 0.5 μl of the lysate (1.2 x 10^5^ GE) obtained from the transfected pupae. After infection, the mean viral load of rDWV was 2.0 x 10^10^ GE/bee in the second and 2.5 x 10^10^ GE/bee in third passage. In parallel, we passaged the DWV-A 1414 isolate (wtDWV) using 0.5 μl of the described crude pupa lysate. A mean viral load of 8.2 x 10^10^ GE/bee was obtained from passage three of wtDWV. Mock infected pupae, which originated from the same combs, were used as controls in these experiments. DWV RNA was not detectable in the mock infected controls. A different RT-PCR assay was performed to differentiate between wtDWV and rDWV. The RT-PCR amplicons from rDWV transfected and rDWV infected bee pupae were completely fragmented after BamHI digest (488 and 276 bp) confirming the rescue of recombinant virus and the absence of field virus replication (Fig 5A and 5B). Western blot analysis of pupae infected with the field virus using the VP1 specific mAb DWVVP1A1 revealed large amounts of mature VP1 (46 kDa) and of an unknown 19 kDa protein, while only a weak signal of VP1 and of the 19 kDa protein was found after transfection of rDWV RNA (Fig 6A). However, a strong VP1 expression was observed after rDWV infection of pupae in subsequent passages. Interestingly, the mAb DWVVP1B1 showed no reactivity with the 19 kDa protein (Fig 6B).

### 3.3. Pathogenicity of rDWV A1414 and wtDWV A1414

Pathogenicity of rDWV and wtDWV was assessed after infection of ten bee pupae with 5 x 10^6^ GE each, compared to a mock-infected control group. The surviving bees from each group at day 22 of development together with the dead pupae withdrawn prematurely from the experiment are presented in a supporting information file (S1 Fig). From the mock-infected control group, one bee pupa died at day two after injection and one bee displayed severe wing deformations after emergence. Eight of the bees appeared healthy with clear unfolded wings (Fig 4B1). In the wtDWV group, six bee pupae died at day three and two bees died at day four post infection (Fig 4B2), giving a mortality rate of 80%. One of the wtDWV infected bees emerged with severely malformed, damaged wings and one showed dwarfism with discoloration (morbidity rate of 100%). One bee of the rDWV infected group died at day three post infection, eight bees emerged with malformed wings, and one bee showed no signs of disease (Fig 4B3), yielding a mortality rate of 10% and a morbidity rate of 90% for rDWV.

### 3.4. Host cell tropism of rDWV and wtDWV A1414

**Fig 7:**
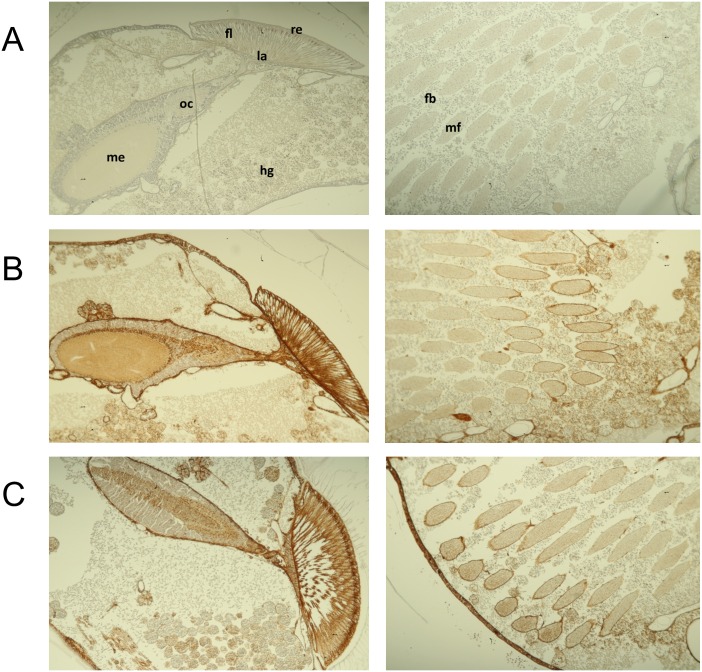
Immunohistochemistry staining of DWV infected cells. Bee pupae were probed using mAb DWVVP1B1 at day three post infection. The head (left row) and thorax region (right row) of mock (A), wtDWV (B) and rDWV (C) infected bee pupae is shown. A broad infection pattern is revealed by VP1 staining for wtDWV and rDWV. Anatomical details are indicated as retina (re), fenestrated layer (fl), lamina (la), outer chiasm (oc), and medulla (me) of the optical lobe and hypopharyngeal glands (hg) for the head in A. In the thorax section, muscle bundles (mb) separated by fat body cells (fb) are marked.

Bee pupae infected with passage three of rDWV and wtDWV were analyzed by immunohistochemistry (IHC) at day three post infection (day 19 of development). Mock-infected control pupae were included as negative controls. The mAb DWVVP1B1 showed no reactivity in the negative control demonstrating the specificity of the staining for the DWV VP1 protein (Fig 7A). Tissue distribution of IHC signals for rDWV-infected bees was similar to that of wtDWV-infected bees (Fig 7B and 7C). DWV infection affected all parts of the bee body, including head, thorax, and abdomen as previously described [15]. In the head, DWV VP1 antigen was found in ocular cells, central nervous system and glandular tissues. In the thorax, connective tissue cells and glands were stained. No VP1 of DWV was found in haemocytes and muscle cells, suggesting that these cells are less susceptible to DWV-A infection.

### 3.5. Electron microscopy of rDWV

**Fig 8:**
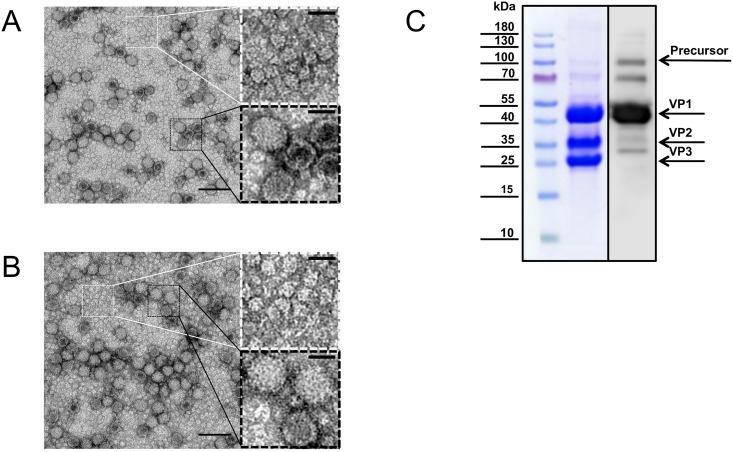
Virions of rDWV (A) and wtDWV (B) visualized by transmission electron microscopy. Proteinacious aggregates (white box) and single virions (black box) are enlarged for both preparations. The bar is 100 nm in the left and 25 nm in the right panels. (C) The protein content of purified rDWV virions is presented after SDS-PAGE in Coomassie stain and Western blot (DVWVP1A1). The apparent bands of DWV structural proteins are indicated.

Virions formed after transfection of rDWV RNA were assessed by transmission electron microscopy. Spherical particles with diameters of about 30 nm were observed in wtDWV (Fig 8A) and rDWV (Fig 8B) concentrates, as previously described for DWV [28]. In these virus preparations, very dense protein aggregates occurred, which most likely consist of DWV structural proteins. The protein composition of the preparations was further analysed by SDS-PAGE and Western blot analysis (Fig 8C), showing three clear bands representing the major DWV structural proteins.

## 4. Discussion

Typical wing deformities, brood losses and colony mortality are observed following varroa mite infestation of European honey bees. These symptoms are indicative of a DWV infection; however DWV is frequently detected in healthy colonies at the same time. The exclusion of additional pathogens responsible for colony mortality is excessively difficult, taken the lack of classical virological tools into account. The same applies for laboratory experiments reproducing the pathogenicity of DWV, in which certain requirements of Koch’s postulates were already fulfilled [15]. Robert Koch defined strict criteria for the establishment of a causative relationship between a microbe and a disease, first published in 1884 [42]. These strict criteria include the isolation of a microbe from a diseased host, its growth in pure culture and the reproduction of the disease in a healthy host after experimental inoculation. Furthermore, the microbe must be re-isolated from the diseased experimental host and identified as being identical to the cultured microbe. Multiple attempts have been made to adapt Koch’s postulates to chronic diseases [43], sequenced-based identification of pathogens [44], general association of environmental factors and disease [45], or the role of genes and their products in pathogenesis [46]. Pure virus cultures depend on biological cloning methods in defined cell culture systems, which have yet to be established for honey bee viruses. In this study, we applied molecular cloning to generate a pure DWV inoculum to better fulfill Koch’s postulates.

Each molecular approach to test candidate genetic factors involved in microbe pathogenesis requires an infection model and the ability to genetically manipulate the microbe. The ability to genetically manipulate the infectious agent is pivotal for controlled investigations of RNA viruses [47]. Infectious clones exist for a large number of positive-stranded RNA viruses, including picorna-, alpha-, pesti-, arteri-, and coronaviruses with genomes up to 30 kb. Reverse genetics has been a key technique for elucidating viral pathogenesis and the functions of viral gene products [48]. Here, we present the first plasmid based reverse genetics system for a DWV-A genome. Key feature of this DWV-A clone is the identity of the 5’-end that exceeds previous full-length DWV sequences by 10 to 21 nucleotides. Sequence comparison to other members of the family Iflaviridae demonstrates a high diversity of the 5’-terminus [49]. Preliminary bioinformatics analyses suggest a stem loop structure downstream 10 unpaired nucleotides at the very 5’end. Further experiments are needed to unravel function and two-dimensional structure of the 5’-NTR. It is noteworthy that the plasmid based copy of the DWV genome originated from a full length PCR product. This approach not only simplifies the construction of the cDNA clone but also warrants that not different—possibly incompatible—fragments of the DWV quasispecies cloud are joined.

The eponymous clinical sign of DWV-A infection of bee pupae is the atrophy or misfolding of the wings, which is caused by vector borne DWV transmission [15, 50]. The unfolding and stretching of the wings represents the first step of a bee’s development after molting to an imaginal stage in their holometabolous metamorphosis. Multiple factors might impair the sensitive process of hymenoptera wing development. Unsuitable incubation conditions, pupal injuries, the lack of a cocoon, hormonal disorders [51] and intoxication [52] have been shown to result in the emergence of adult bees with crippled or malformed wings. We were able to reproduce the typical clinical signs of DWV infections using synthetic RNA derived from a plasmid clone of DWV-A. Pathogenicity of the DWV-A clone was assessed by a high titer infection experiment showing a morbidity rate of 90%. In contrast, equivalent infections with the field isolate DWV-A 1414 caused a morbidity of 80%. Differences in the morbidity rate might be due to a clonal infection in case of the recombinant virus, compared to an infection with a cloud of divergent genomes in case of the field virus isolate. The importance of quasispecies for DWV pathogenesis has been put forward in previous studies reflecting the natural situation in most RNA viruses [27]. On these grounds it is exciting that a single genome from the quasispecies cloud is able to reproduce disease. We now can study the speed, complexity and direction of the development of DWV-A quasispecies radiating from a single clone. Further to this, virulence factors, or more likely, virulence associated gene alterations can be investigated in detail.

Until now, it is not known whether wing deformities arise from wing tissue infection or result from systemic damages. Our immunohistochemistry data underlines that DWV injection led to systemic infections affecting all parts of the bee’s body. DWV-A was detected in bee heads within the neural and gland tissues, as previously described [29]. In nurse bees, the exocrine glands of the head secrete the royal jelly. Since signals of DWV VP1 were detected in all secretory glands of the head, an evolutional adaptation of DWV to oral transmission is very likely [53]. Nutrient exchange is a continuous process in the bee colony that might contribute to transmission, horizontal infection and persistence of DWV in honey bee colonies [15]. The application of the reverse genetics system will provide a new basis to study the factors of DWV host cell tropism and to investigate the molecular mechanisms behind DWV transmission.

## Supporting Information

S1 Fig Pathogenicity of wtDWV and rDWV. Injection of ten bee pupae with PBS (A), 5 x 10^6^ GE of wtDWV (B), and 5 x 10^6^ GE of rDWV (C) was performed at day 15 of development. Outcome of the infection experiment documented at day 22 of development.
(TIF)
