# Supplementary figures and images for: Construction and Rescue of a Molecular Clone of Deformed Wing Virus (DWV)
Source: PLoS One. 2016 Nov 9;11(11):e0164639. doi: 10.1371/journal.pone.0164639 (PMC5102418; doi:10.1371/journal.pone.0164639)

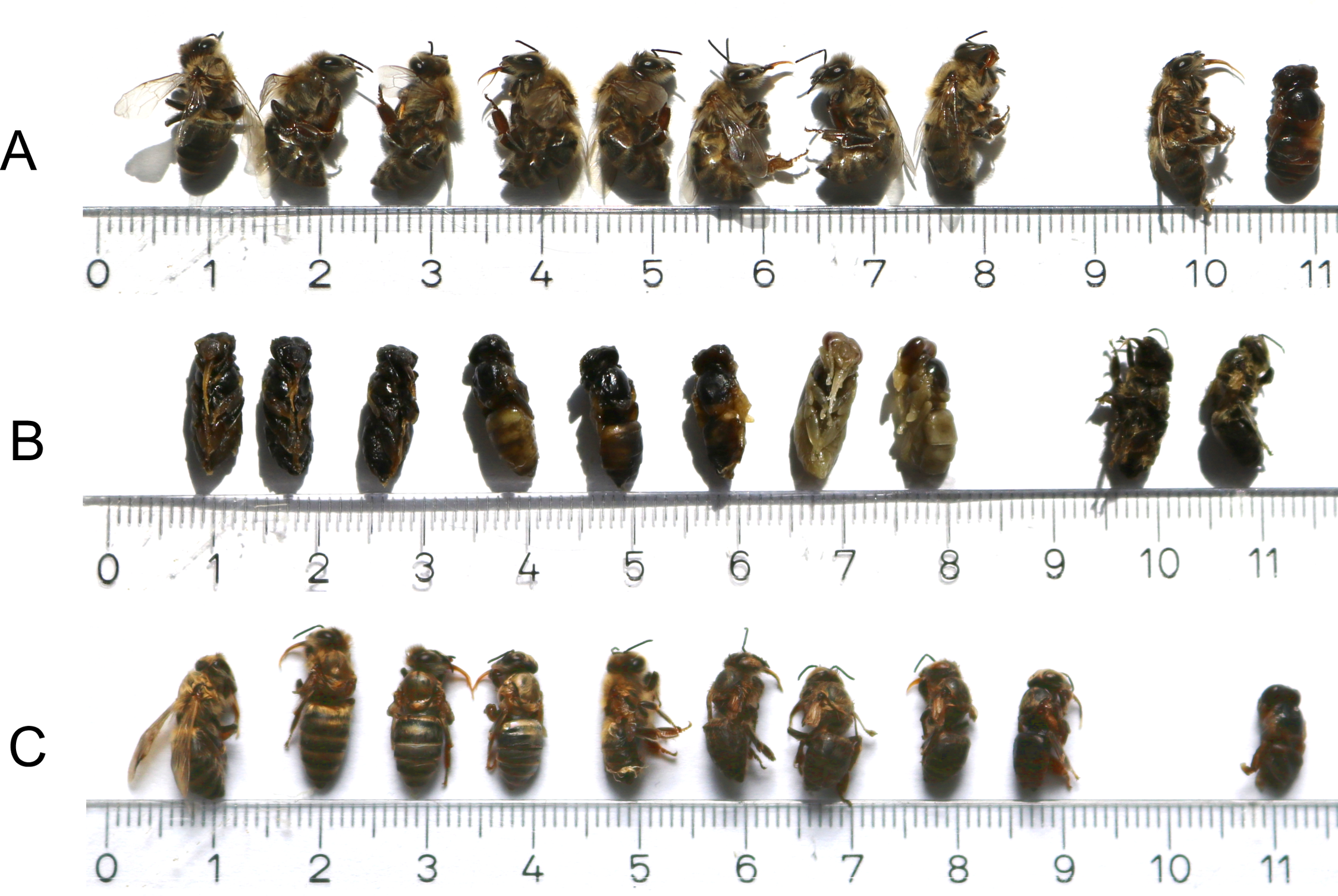

Supplement: S1 Fig — Injection of ten bee pupae with PBS (A), 5 x 10^6^ GE of wtDWV (B), and 5 x 10^6^ GE of rDWV (C) was performed at day 15 of development. Outcome of the infection experiment documented at day 22 of development. (TIF) [file pone.0164639.s001.tif]
